# Novel Heterozygous Missense Variant in GRIA4 Gene Associated With Neurodevelopmental Disorder With or Without Seizures and Gait Abnormalities

**DOI:** 10.3389/fgene.2022.859140

**Published:** 2022-04-20

**Authors:** Hua Wang, Jiatong Liu, Fuwei Li, Ziteng Teng, Mingyu Liu, Weiyue Gu

**Affiliations:** ^1^ Department of Pediatric Neurology, Shengjing Hospital of China Medical University, Shenyang, China; ^2^ Chigene (Beijing) Translational Medical Research Center Co., Ltd., Beijing, China

**Keywords:** GRIA4, NEDSGA, neurodevelopmental disorder, trio-whole exome sequencing, novel heterozygous missense variant

## Abstract

**Objective:** Neurodevelopmental disorder with or without seizure and gait abnormalities (NEDSGA, MIM * 617864) is a newly described autosomal dominant inherited disease caused by a heterozygous variant in the GRIA4 gene. *GRIA4* plays an essential role in excitatory synaptic transmission. In this study, we presented the clinical and genetic features of a female patient carrying a novel *de novo* variant in *GRIA4* and further reviewed the previously reported five different patients.

**Methods:** Evaluation of the patient included a detailed history and clinical examination. Trio-whole exome sequencing (WES) was performed to identify pathogenic variants in NEDSGA. Sanger sequencing was further used to validate the variants.

**Results:** We described the clinical features of an infant diagnosed with NEDSGA caused by a *GRIA4* variant, who presented with severe developmental delay, limb hypertonia, generalized seizure, retinal hypoplasia, and chorioretinal hyperpigmentation. The patient developed tricuspid regurgitation, and imaging examination revealed a patent foramen ovale. Trio-WES identified a novel *de novo* heterozygous missense variant c.1918G>T, p.Ala640Ser in the GRIA4 gene. Multiple in silico tools predicted deleterious effects of p.Ala640Ser.

**Conclusion:** A novel heterozygous missense variant in the GRIA4 gene (c.1918G>T) identified in the proband expanded the genotypic and phenotypic spectrum of disorders associated with *GRIA4* variants. This is the first NEDSGA case reported in China. Our findings provide valuable information for the differential diagnosis of neonatal onset neurodevelopmental disorders.

## Introduction

Neurodevelopmental disorder with or without seizure and gait abnormalities (NEDSGA, MIM * 617864) is an early onset of neurodevelopmental disorder associated with global developmental delay and variable intellectual disability. Most patients presented with irritability, stiffness, seizure, and hypertonia early in life, followed by spasticity and impaired gait. NEDSGA is caused by the gene *GRIA4* (MIM * 138246) located on chromosomes 11q22 and is inherited in an autosomal dominant manner. GRIA4 encodes glutamate ionotropic receptor AMPA type subunit 4 and plays an essential role in excitatory synaptic transmission. *GRIA4* is ubiquitous in the central nervous system and is highly expressed in the thalamus, especially in the thalamic reticular nucleus (Beyer et al., 2008). GRIA4 in the rat brain is relatively high in CA1 pyramidal cells, hippocampal dentate gyrus, cerebral cortex, and cerebellar granule cells (Keinänen et al., 1990). Since Martin first reported NEDSGA in 2017 (Martin et al., 2017), only five NEDSGA patients with *GRIA4* variants have been reported, ranging in age from 4 to 21 years. All *GRIA4* variants described are *de novo* heterozygous missense variants that cluster in the transmembrane and ligand-binding domains of the GRIA4 protein.

In this study, we reported a 9-month-old girl from a nonconsanguineous family with healthy parents. The patient presented with severe global developmental delay (HP:0011344), limb hypertonia (HP:0002509), partial seizure (HP:0007359), retinal hypoplasia (HP:0007770), chorioretinal hyperpigmentation (HP:0040031), tricuspid regurgitation (HP:0005180), and patent foramen ovale (HP:0001655). Using trio-whole exome sequencing (WES), we identified a novel *de novo* heterozygous missense variant in *GRIA4*, c.1918G>T, p.Ala640Ser. To the best of our knowledge, this is the first NEDSGA case reported in China. Our finding expands the genotypic and phenotypic spectrum associated with *GRIA4*.

## Patients and Methods

### Patients

The Ethics Committee of the Shengjing Hospital of China Medical University approved this study. The patient’s legal guardians signed the informed consent for the study.

The proband was a 9-month-old girl, the first child of healthy Chinese parents. The mother was 34 years old, and the father was 40 years old. The patient was transferred to our hospital due to the occurrence of breathing difficulties, limb hypertonia, and seizure. Electroencephalogram (EEG) and brain magnetic resonance imaging (MRI) were performed on her clinical presentation.

### Variation Analysis

DNA was obtained from the peripheral venous blood of the girl and the parents and submitted to the Chigene Translational Medicine Research Center Co., Ltd., Beijing, for trio (parents and proband)-WES. Whole-exome capture was xGen Exome Research Panel v2.0 (IDT, Iowa, United States). The sequencing operation flow was standardized on the DNBSEQ-T7 (BGI, China) platform. Raw-sequencing reads were processed by fastp (https://github.com/OpenGene/fastp) for adapter removal and low-quality read filtering. High-quality sequencing data were generated and performed on the Ensemble GRCh37/hg19 reference genome using the Burrows-Wheeler Aligner (BWA, https://github.com/lh3/bwa). GATK (http://www.broadinstitute.org/gatk/) was used for base quality score recalibration and SNP and INDEL calling. Trio-WES had a mean depth of coverage of at least 172× per sample, with 98% of the exome covered 20× or greater. The sequencing depth ranged from 192×−413× coverage of the GRIA4 gene, with 100% target region coverage >10× sequencing depth. Pathogenicity of the genetic variants were predicted by bioinformatics tools such as PolyPhen (http://www.bork.embl-heidelberg.de/PolyPhen/), Mutation Taster (http://www.mutationtaster.org), REVEL (https://sites.google.com/site/revelgenomics/), and CADD (http://cadd.gs.washington.edu/). Finally, the variants were classified according to the American College of Medical Genetics and Genomics (ACMG) guidelines (Richards et al., 2015).

Pathogenic variants were detected using trio-WES, followed by Sanger sequencing validation. Primer 5.0 primer software was used to design the *GRIA4* primers: forward primer (5′- GCT​AAA​GCC​CAT​GGT​ATA​ATT​GTT​G-3′) and reverse primer (5′-GCA​TGG​TGA​ATT​GAC​GGT​ATT​TCT​T-3′). Sanger sequencing was further performed using the 3730xl DNA Analyzer (Applied Biosystems, United States). The identified rare variants have been submitted to the ClinVar database (https://www.ncbi.nlm.nih.gov/clinvar/) (Accession number: SCV002055968).

The GRIA4 protein is highly conserved among vertebrates, showing 86% sequence identity among 10 different species, including primates, rodents, laurasiatheria, placental mammals, sauropsida, and fish (http://asia.ensembl.org/index.html). Multiple protein sequence alignments were conducted on MEGA X (Kumar et al., 2018). 3D modeling of structural effects was performed using the GRIA4 protein structure (AlphaFold, AF-P48058-F1) (Varadi et al., 2022). The models were visualized using Pymol (www.pymol.org) (Schrodinger, 2015).

## Result

### Clinical Features

**FIGURE 1:**
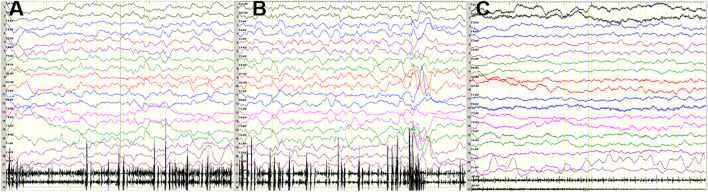
**(A, B)** At the age of 3 months, sleep EEG of the patient revealed multifocal sharp waves and low waves. **(C)** Wake EEG was normal.

The girl was born by an uncomplicated delivery at 38 weeks’ gestation, with a normal birth weight of 3,200 g and a body length of 50 cm. Neonatally she presented with irritability, breathing difficulties, limb hypertonia, and partial seizure. Cardiac ultrasonography showed tricuspid regurgitation and patent foramen ovale. Fundus examinations revealed hypoplasia of the retina and chorioretinal hyperpigmentation. Brain magnetic resonance imaging (MRI) was normal. Her electroencephalogram (EEG) demonstrated multifocal sharp waves and low waves during sleep (Figures 1A–C). Laboratory tests showed hyperlactemia and hyperammonemia.

In the follow-up, she suffered from severe developmental delay. At 4 months, she started taking lysine, inosite and vitamin B12 oral solution, GABA compound nutritious solid drink, and cerebroprotein hydrolysate oral solution. Limb hypertonia persisted, but seizures were well controlled.

To the best of our knowledge, only one publication (Table1) (Martin et al., 2017) reported a total of five cases with *GRIA4* variants. Our patient is the sixth case of NEDSGA, in which neurodevelopmental disorders with seizures and abnormal gait were the most common phenotypes caused by pathogenic variants in *GRIA4*.

**TABLE 1:** Clinical features of individuals with *de novo GRIA4* variants.

Patient	1 (This study)	2 (Martin et al., 2017)	3 (Martin et al., 2017)	4 (Martin et al., 2017)	5 (Martin et al., 2017)	6 (Martin et al., 2017)	Total
Variant	c.1918G>T,p.Ala640Ser	c.1915A>T,p.Thr639Ser	c.1921A>G,p.Asn641Asp	c.1928C>G,p.Ala643Gly	c.1931C>T,p.Ala644Val	c.2090G>C,p.Arg697Pro	—
Provean	Deleterious (-2.68)	Deleterious (−3.57)	Deleterious (−4.47)	Deleterious (−3.58)	Deleterious (−3.58)	Deleterious (−2.69)	—
SIFT	Damaging (0.014)	Damaging (0.001)	Damaging (0.001)	Damaging (0.001)	Damaging (0.0)	Damaging (0.005)	—
PolyPhen	Probably damaging (1.0)	Probably damaging (1.0)	Probably damaging (1.0)	Probably damaging (1.0)	Probably damaging (1.0)	Benign (0.026)	—
Mutation Taster	Disease_causing (1)	Disease_causing (1)	Disease_causing (1)	Disease_causing (1)	Disease_causing (1)	Disease_causing (0.985836)	—
CADD	Deleterious (26.3)	Deleterious (24.7)	Deleterious (26.3)	Deleterious (29.2)	Deleterious (29.8)	Deleterious (24.1)	—
Gender	Female	Male	Male	Male	Male	Female	—
Age at last exam	9 months	15 years	21 years	4 years	4 years	4 years	—
Seizures	Partial seizures	No	Intractable generalized seizures	Seizure-like episodes	Febrile seizures	No	—
Age at epilepsy onset	1 day	—	5 weeks	14 months	13 months	—	—
Seizure outcome	Seizure control	—	Refractory	Seizure control	Seizure control	—	—
EEG	Multifocal sharp waves, low waves during sleep	Unremarkable	Diffuse cerebral disturbance without electrographic correlates to the seizures	Generalized slowing, no epileptiform discharges	Generalized spikes and waves during sleep	Unremarkable	4/6
Brain MRI	Unremarkable	Unremarkable	Bilateral symmetric extensive atrophy of frontal lobes, mild frontal ventriculomegaly, thin corpus callosum	Optic nerve hypoplasia	Unremarkable	Unremarkable	2/6
Muscle	Hypertonia	Hyperekplexia with exaggerated head-retraction reflex, stiffness, and hypertonia	Severe spastic quadriplegia and hypertonia with contractures	Spasticity	Mild muscular hypotonia (neonatal)	Unremarkable	5/6
Developmental delay	Moderate to severe	Mild to moderate	Severe	Severe	Moderate to severe	Mild to moderate	6/6
Motor development	Too young to evaluate	Clumsy or stiff gait	Inability to walk	Clumsy or stiff gait	Clumsy or stiff gait	Normal	4/5
Speech impairment	Too young to evaluate	Speech with dysarthria	Absent speech	Absent speech	Absent speech	Poor speech	5/5
Eye features	Hypoplasia of the retina, chorioretinal hyperpigmentation	NA	Strabismus	Nystagmus, optic nerve hypoplasia	NA	NA	3/3
Heart	Tricuspid regurgitation, patent foramen ovale	NA	NA	NA	NA	NA	1/1
Additional features	Hyperlactemia, hyperammonemia	Sleeping problems, dysmorphic features	Feeding difficulties, apneas, choreiform movements, dysmorphic features	Dysmorphic features	Stereotypic hand movements	Hyporeflexia, simian crease on both hands	—

EEG, electroencephalograph; MRI, magnetic resonance imaging; NA, not available.

All six patients (2 females and 4 males, aged 9 months to 21 years) had neurodevelopmental disorders, with mild (2/6; 33%) to severe (4/6; 67%) developmental delay. Four patients (4/6; 67%) had movement disorders, including clumsy or stiff gait and inability to walk. Three patients (3/6; 50%) presented with ocular anomalies, including strabismus, nystagmus, optic nerve hypoplasia, hypoplasia of the retina, and chorioretinal hyperpigmentation. Five patients (5/6, 83%) had poor speech or aphasia. Seizures occurred in four of six patients (67%). Patient 2 had sudden muscle cramps/seizures lasting up to 1 hour after trauma, while patient 6 had no seizures. The age of seizure onset ranged from 1 day to 14 months, with a median age of 7 months. Of note, seizure disorders were found in all patients with severe developmental delay. A broad spectrum, including generalized seizures, febrile seizures, and seizure-like episodes, was reported. Three patients (3/4; 75%) achieved seizure control by therapies. However, patient 3 developed refractory seizures and status epilepticus. All four patients with seizures exhibited abnormal EEG and two of them had abnormal MRI.

Our patient presented with severe developmental delay, limb hypertonia, partial seizure, retinal hypoplasia, chorioretinal hyperpigmentation, tricuspid regurgitation, and patent foramen ovale, whereas the girl had no signs of craniofacial or MRI abnormalities. In addition, our patient was too young to assess her ability to walk or speak. Together with our clinical findings, half of the affected patients had ocular abnormalities, so the patient’s ocular examination should not be ignored.

### Variation Analysis

**FIGURE 2:**
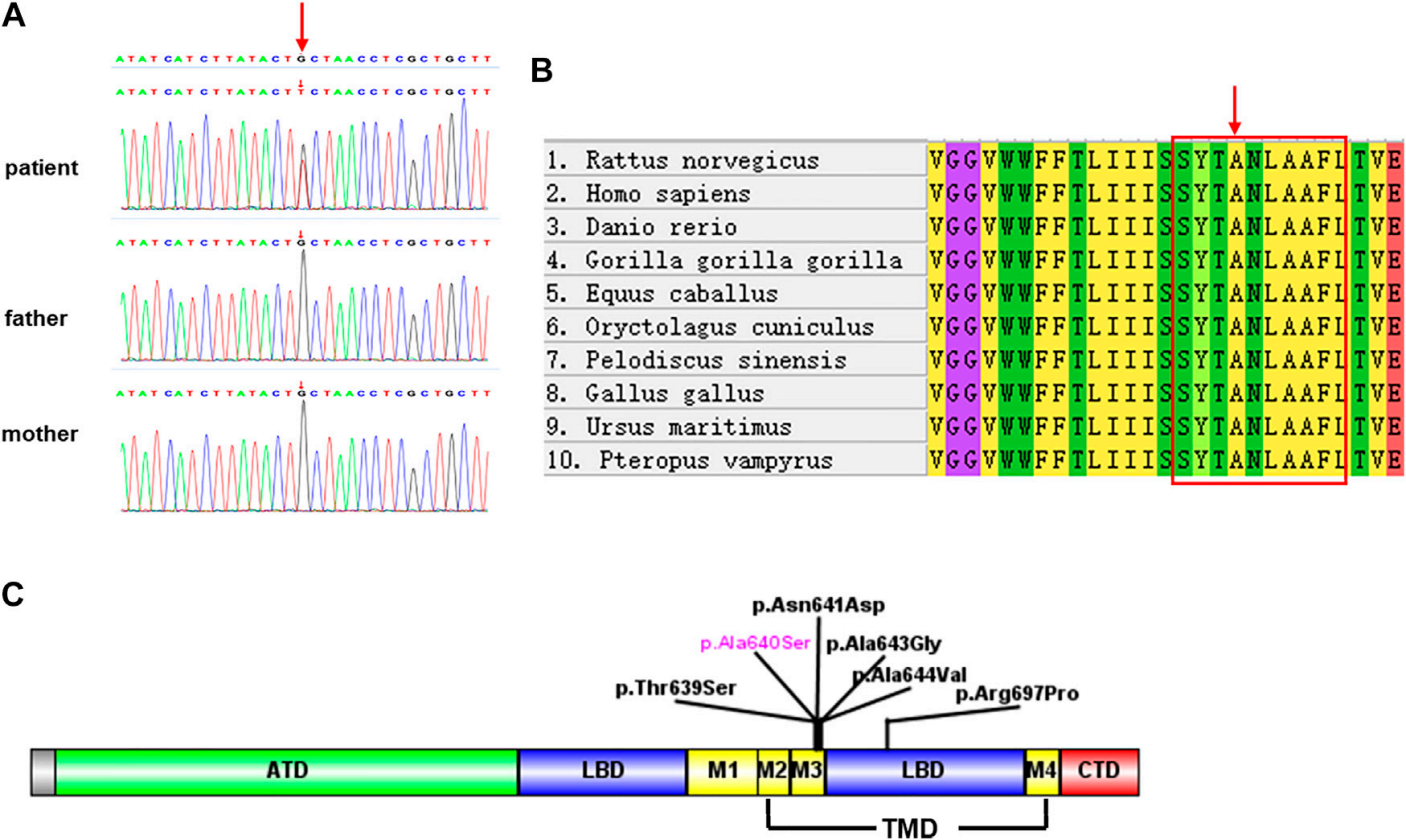
**(A)** Sanger sequencing validation of the variant c.1918G>T in the proband and parents. **(B)** Multiple species sequence alignment. The mutated alanine A640 (red arrow) falls within a highly conserved SYTANLAAF motif (red box). **(C)** Schematics depicting the location of GRIA4 variants. Amino-terminal domain (ATD, in green), ligand binding domain (LBD, in blue), transmembrane domain (TMD, in yellow), and carboxyl-terminal domain (CTD, in red). TMD contains three transmembrane domains (M1, M3, and M4) and re-entrant membrane loop M2.

Trio-WES identified a *de novo* heterozygous variant in *GRIA4* in the patient: chr11:105797537G>T(hg19), c.1918G>T transition. The c.1918G>T is predicted to result in the substitution of the alanine residue p.Ala640Ser. This variant has not been reported in public databases (gnomAD v2.1.1, http://gnomad.broadinstitute.org/ and the 1000 Genomes Project, http://www.internationalgenome.org). In addition, it was predicted as pathogenic by multiple bioinformatic tools (SIFT: damaging; PolyPhen: probably damaging; Mutation Taster: disease_causing; REVEL: deleterious; CADD: 26.3). According to the ACMG guidelines, we confirmed the variant to be pathogenic (PS2+PM1+PM2+PP2+PP3) (Richards et al., 2015; Harrison et al., 2019). Furthermore, we did not detect pathogenic or likely pathogenic variants in genes known to be associated with neurodevelopmental disorder in probands using Trio-WES. The variants of the *GRIA4* gene were confirmed by Sanger sequencing (Figure 2A).

**FIGURE 3:**
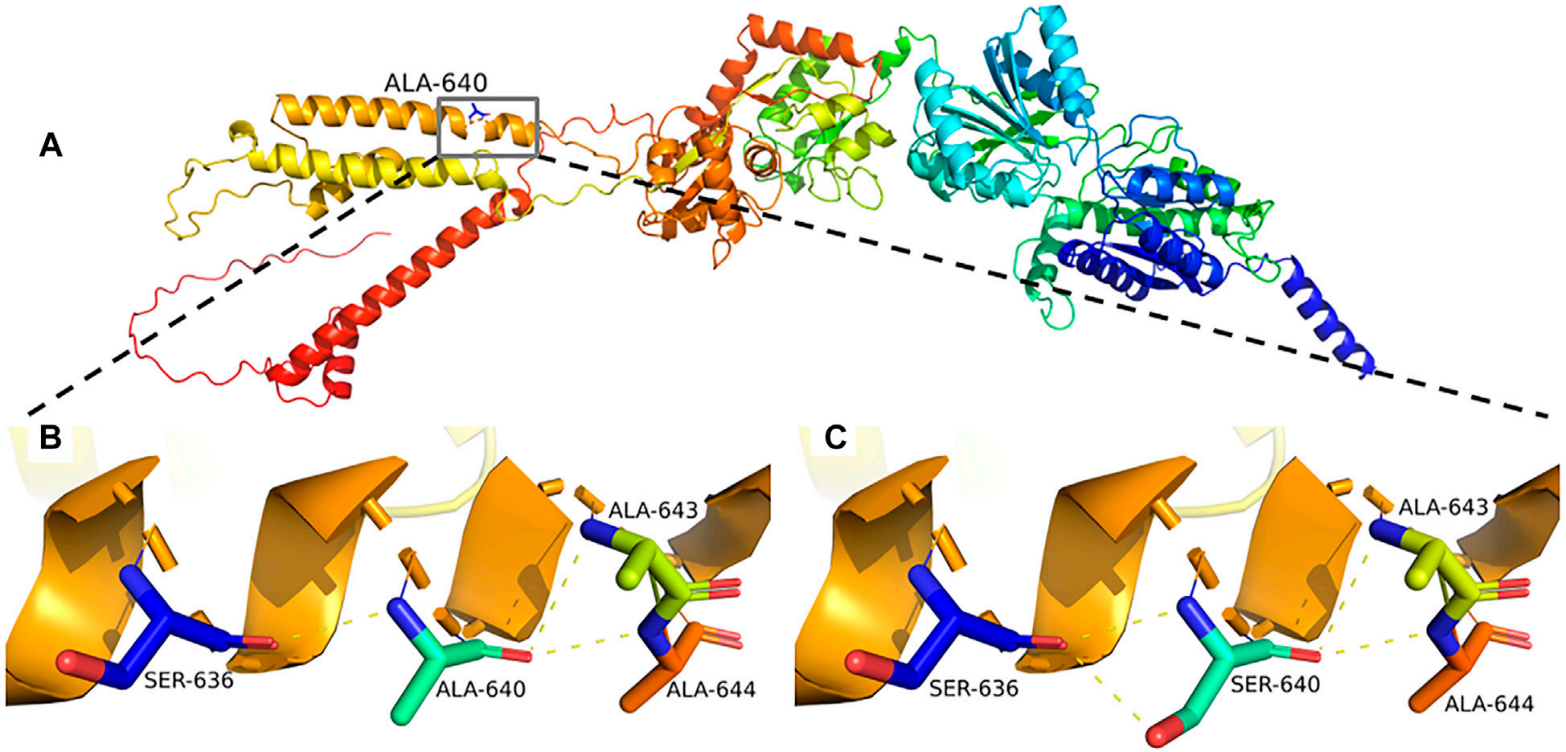
**(A–C)** Predicted mutational impact of p.Ala640Ser on the GRIA4 protein structure. Compared to the WT model **(B)**, the new hydrogen bond formed between the mutant Ser640 **(C)** and the neighboring Ser636 hydrogen bonds, yellow dashed lines.

Sequence alignment among multiple vertebrate species suggested that p.Ala640Ser was located at a highly conserved site (Figure 2B) and in the transmembrane domain (Figure 2C). 3D structural analysis of the *GRIA4* protein showed that the mutated Ser640 residue formed a new hydrogen bond between Ser640 and the neighboring Ser636 compared to the WT model (Figures 3A–C). The mutation of Ala640Ser, located in an alpha-helix, causes the change of hydrogen bond between residues, and further affects protein folding.

## Discussion

NEDSGA is a newly recognized rare neurodevelopmental disorder caused by a heterozygous variant in the *GRIA4* gene. All the reported *GRIA4* variants were heterozygous missense, located in the transmembrane and ligand-binding domains (Figure 2C) (Martin et al., 2017).

AMPARs (*α*-amino-3-hydroxy-5-methyl-4-isoxazolepropionate receptors) consist of four subunits GluR1-GluR4, mediate fast excitatory neurotransmission in the central nervous system, and play a critical role in learning, memory formation, and brain development. Each iGluR subunit comprises an amino-terminal domain (ATD), a ligand-binding domain (LBD), a transmembrane domain (TMD), and a carboxyl-terminal domain (CTD) (Figure 2C) (Traynelis et al., 2010; Sobolevsky, 2015). TMD contains three transmembrane domains (M1, M3, and M4) and re-entrant membrane loop M2 (Twomey et al., 2017). M3 transmembrane segment contains a nine amino acid sequence, SYTANLAAF motif, that plays a crucial role in channel activation and gating, and is highly sensitive to a conservative amino acid change (Salpietro et al., 2019). The variant p.Ala640Ser is located in the SYTANLAAF motif that is highly conserved throughout all members of the glutamate receptor family (Jones et al., 2002). Furthermore, multiple sequence alignment analysis revealed that p.Ala640 is highly conserved in different species (Figure 2B). For the variant p.Ala640Ser, the presence of the serine acid residue is predicted to lead to the formation of a new hydrogen bond with the p.Ala636 residue. p.Ala640Ser is in hydrophobic membrane-spanning helix M3, mutation of a critical hydrophobic residue (Ala) to a hydrophilic one (Ser) is predicted to destroy the hydrophobic interactions and further disrupt the gating mechanism (Sobolevsky et al., 2003; Sobolevsky, 2015). In the previous study, Salpietro et al. identified the variant p.Ala639Ser in *GRIA2* in two unrelated infants with uncontrolled seizures from the first days of life (Salpietro et al.). And the variant p.Ala639Ser in *GRIA2* is located in the same position (in the motif of SYTANLAAF) as the variant p.Ala640Ser in *GRIA4* in our patient. An *in vitro* functional study showed that the variant p.Ala639Ser in *GRIA2* reduced agonist-induced current amplitude, resulting in a significantly reduced cell-surface expression of *GRIA2* (Salpietro et al.). Therefore, we speculated that the variant p.Ala640Ser in GRIA4 had a high probability of pathogenicity, as it was also located in the same important functional domain. Moreover, further investigation by the electrophysiology experiments and biotinylation assay will be necessary to determine whether p.Ala640Ser in GRIA4 affects the channel synthesis or trafficking and its effect on currents.

In conclusion, using trio-WES, we identified a novel *de novo* heterozygous missense variant in the *GRIA4* gene and diagnosed the sixth NEDSGA patient with severe developmental delay, limb hypertonia, partial seizure, hypoplasia of the retina, chorioretinal hyperpigmentation, and other clinical characteristics. Our findings enriched the phenotypic spectrum of genetic disorders associated with *GRIA4* variants. And genetic evidence further supports the association of rare and newly reported NEDSGA caused by the *GRIA4* gene. Our data will be helpful in diagnosing NEDSGA, especially in the affected newborns.

## Data Availability

The datasets presented in this study can be found in online repositories. The names of the repository/repositories and accession number(s) can be found at: https://www.ncbi.nlm.nih.gov/, SCV002055968.
